# A prototype system for the removal of ^137^Cs from liquid radioactive waste using reverse osmosis membrane

**DOI:** 10.1007/s11356-024-33426-3

**Published:** 2024-05-02

**Authors:** Mohamed Shaltout, Shaban Kandil, Abdou Saad El-Tabl, Hany Aglan, Ahmed M. Shahr El-Din, Yasser T. Mohamed

**Affiliations:** 1 Analytical Chemistry and Control Department, Hot Labs. and Waste Management Center, Egyptian Atomic Energy Authority, Cairo, Egypt; 2 Labeled Compounds Department, Hot Labs. Center, Egyptian Atomic Energy Authority, Cairo, Egypt; 3 Cyclotron Facility, Nuclear Research Centre, Egyptian Atomic Energy Authority, Cairo, Egypt; 4 Chemistry Department, Faculty of Science, Menoufia University, Shibîn El Kôm, Menoufia, Egypt

**Keywords:** ^134,137^Cs, Reverse osmosis membrane, Removal, EDTA, Citric acid, Elution

## Abstract

Cesium removal from aqueous solutions of radioactive waste streams is a challenge in the field of radioactive waste management; this is due to the small atomic radii of Cs^+^ metal ions and their high migration ability. So, the development of a withstand system for the removal of Cs^+^ is crucial. In the current study, the removal of radioactive cesium from aqueous solutions using an RO-TLC membrane was studied. Two modifications were conducted; the first is to enlarge the cesium metal ion radii by interacting with mono- and dibasic acids, namely, stearic acid, tartaric acid, citric acid, and EDTA, and the second is the modification of the RO membrane pore size via reaction with the same acids. The modification was confirmed using SEM, FTIR, and EDX analysis techniques. The Cs^+^ and K^+^ rejection capacities and water permeability across the membrane at 1.5 bars were evaluated. Along with using the above-mentioned acids, the Cs^+^ metal ion retention index (*R*_Cs_) was also obtained. It was found that employing EDTA as a chelating agent in an amount of 1.5 g/L in conjunction with the variation of feed content since it provided the highest value of *R*_Cs_ ~ 98% when used. Moreover, the elution of Cs^+^ using water, EDTA, ammonia, and HCl is also investigated. The optimal value of the eluent concentration was (0.25 M) HCl. Finally, Langmuir and Freundlich isotherm models were applied for a better understanding of the sorption process. The results of the present work more closely match the Langmuir isotherm model to determine the dominance of the chemical sorption mechanism.

## Introduction

One of the major problems all over the world in the twenty-first century is water contamination (ONU 2012). Recently, there has been a global concern over radioactive pollution in water and the focus has shifted toward the development of advanced decontamination systems to overcome the issue (Munter 2013; Zeitoun 2011). Many conventional water treatment methods have been incorporated to treat water contaminated with heavy metals and radioisotopes such as solvent extraction, sorption, and ion exchange (El-Sadek et al. 2021; Hassan et al. 2019; Mahrous et al. 2022; Mansy et al. 2023, 2017). There are some studies about the influence of conventional treatments applied at water treatment plants to reduce radioactivity (Arnal et al. 2000; Baeza et al. 2006; Commission 2002; Gäfvert et al. 2002; Jiménez and De La Montaña, 2002; Montaña et al. 2013; Munter 2013; Palomo et al. 2010). However, these methods have several limiting factors including thermal instability, the formation of toxic intermediates, and high energy consumption (Clearfield 2010; Dakroury et al. 2020; Wang and Zhuang 2019). A promising use for membrane technology is the efficient removal of heavy metals and radioactive pollutants from aqueous solutions (Arnal et al. 2000; Zakrzewska-Trznadel 2013; Dakroury et al. 2020; Kumar et al. 2020). The advantages of using membrane technology for radionuclide removal include a high decontamination factor (or volume reduction), low energy consumption, and compatibility with existing systems. However, it also has limitations, such as membrane fouling and potential structural changes after radiation exposure (Kakehi et al. 2017). Additionally, the quality of membranes suitable for the treatment of radioactive wastewater, particularly for desalination technology, is still far from adequate. Membrane desalination is the process by which salt and minerals are removed from water solution when they pass through a semipermeable membrane. Membrane techniques used for desalination include forward osmosis (FO), membrane distillation (MD), electrodialysis (ED), and reverse osmosis (RO) (Charcosset 2022). Among all of these techniques, RO has the capability for direct cesium ion retention. At the Radioactive Waste Management Plant in Poland, a three-stage RO unit pilot scale was designed and constructed for the treatment of low and intermediate-level radioactive wastewater (LLW and ILW) (Chmielewski AG. et al., 2001). It was also stated that RO was used to treat LLW from the Fukushima Daiichi nuclear plant disaster (Sylvester et al. 2013). As part of their treatment process, (Arnal et al. 2003) they used RO to remove ^137^Cs (300 kBq/L≈ 8.1 µCi/L) from wastewater. They cleaned more than 90% of the initial volume through membrane treatment and almost 98% through evaporation in the final stage (Arnal et al. 2003). Commercial membranes are available in a wide variety of shapes, including flat sheet, hollow, and spiral coiled, and they have variable pore diameters (Zakrzewska-Trznadel 2001). An RO membrane typically consists of three layers: a separating functional layer, a porous support layer made of polysulfone, and a substrate made of polyester non-woven fabric. Cross-linked aromatic polyamide creates the semipermeable membrane with RO function in the separating functional layer. No RO functions are visible, but the other two layers support the framework that separates the functional layer from operational pressure. As a result, the cross-linked aromatic polyamide’s physical and chemical characteristics determine how well the RO membrane performs (Takao et al. 2012). As membrane technology evolves rapidly, more and more manufacturers are entering this industry and offering high-quality, low-cost commercial membranes. Manufacturers of membranes (Wang and Zhuang 2019) like Dow, LG Chem, General Electric, OSPURA, KOCH, VONTRON, and Pentair offer commercial membranes for purchase. Pentair® TLCTM residential membranes are cutting-edge TLC (Thin Layer Composite) membranes that deliver high-quality water for a variety of water chemistries, according to the manufacturer. It would be able to reject 98% of a 2000 ppm and even a 500 ppm NaCl solution at pressures of up to 7 bar throughout a pH range of 4–11.

The goal of the current study is to remove the radioactive cesium from a liquid radioactive waste by designing a prototype system based on modifying the structure of spiral-wound RO membrane with four selected acids, namely, EDTA, tartaric, stearic, and citric acids.

## Experimental

Cesium chloride (CsCl) and potassium hydroxide (KOH) were provided by Sigma-Aldrich, stearic and tartaric acids by Merck, and citric acid and EDTA by Bio-Rad Laboratories. Double distilled water was employed in all investigations. The real radioactive liquid waste was provided by the RPF Facility, Egyptian Atomic Energy Authority.

### Characterization

The characterization of the RO membrane is conducted using different analysis techniques. Surface morphology, active function groups, and the elemental analysis of the membrane before and after modification were determined by SEM, FTIR, and EDX, respectively.

### Instrumentation

**Fig. 1:**
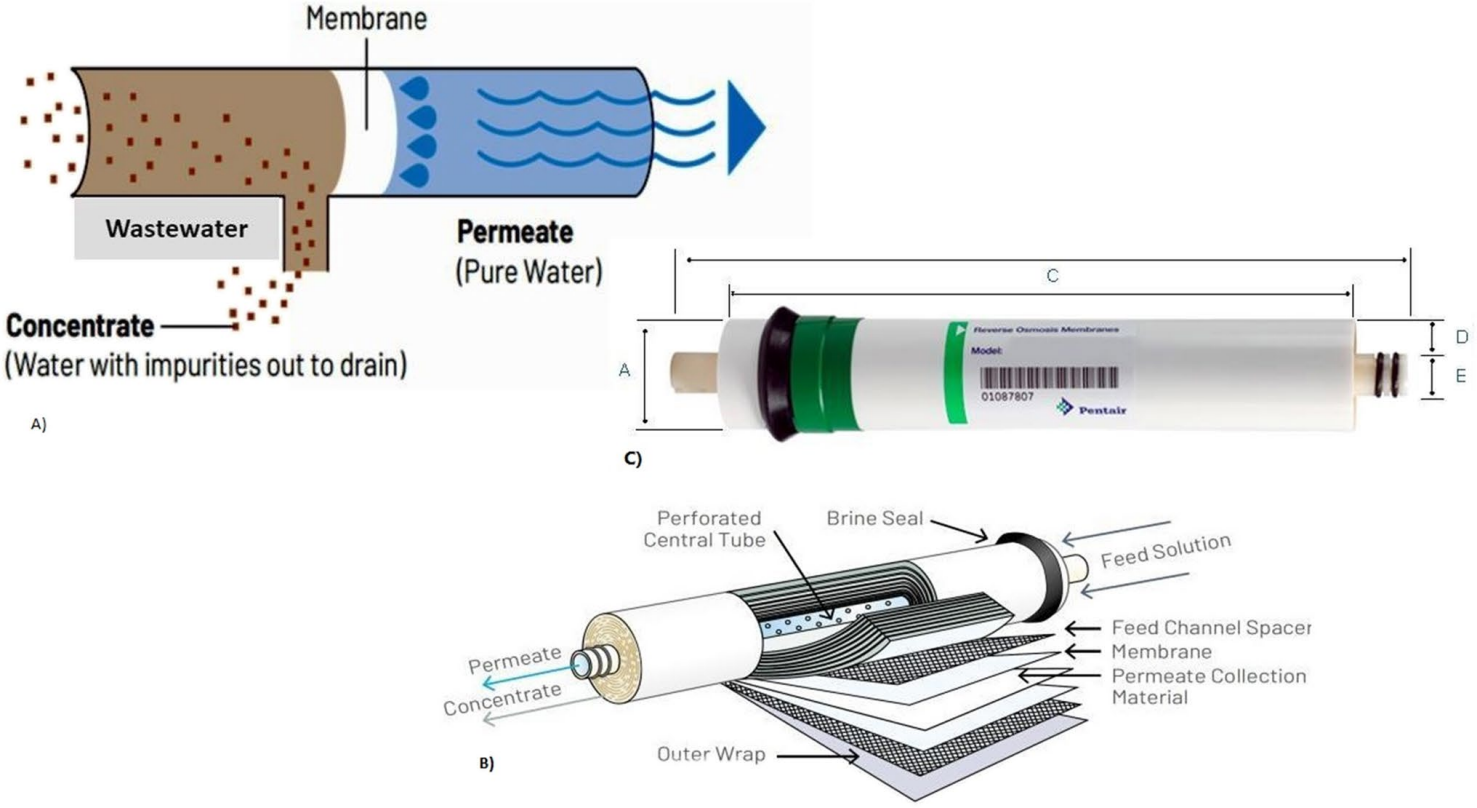
**A** Schematic for the treatment process inside the TLC-75 cartridge. **B** The anatomy of (TLC-75) RO membrane. **C** Dimensions of (TLC-75) RO membrane (A = 4.5, B = 29.85, C = 25.40, D = 2.22, E = 1.72) cm

The reverse osmosis–thin layer composite (RO-TLC) membrane, model (TLC-75, Japan), was purchased from the Egyptian market; full specifications are listed in Table 1 along with its description and dimensions which are presented in Fig. 1. The K^+^ and Cs^+^ metal ion concentrations were measured by the ICP-OES (ULTIMA2 ICP, Jobin Yvon S.A., France). The radionuclide of interest, ^137^Cs, was determined by a high-resolution P-type coaxial High Purity Germanium Detector HPGe, (GX2518 Model, Canberra, USA), with a Pb shield shell coated with a thick copper layer from the inside. A multi-channel analyzer with 16,384 channels, an integrated power supply, and an amplifier is connected to the HPGe detector (Inspector, 2000 Model, Canberra Series, USA). A typical mixed calibration source containing the radionuclides ^152^Eu, ^137^Cs, and ^60^Co was used to calibrate the energy in the low, intermediate, and high gamma energy ranges. To obtain the required efficiency values, efficiency calibration was done using 400 mL double distilled water combined with 0.1 mL standard radioactive material of ^152^Eu in a Marinelli beaker.

**Table 1:** Specifications of the (TLC-75) RO membrane

Specification	Value	Specification	Value
Model	TLC-75	Max. Temp. (°C)	49.0
Flow rate^¥^	75 gpd	pH-range	4–11
Rejection ability^¥^	98%	Hardness	17 °F
Maximum pressure	6.9 bar	Free chlorine	0.1 ppm

^¥^Nominal performances are based on 500 ppm softened tap water at 4.5 bar, 25 °C, and 15% recovery after 24 h. Individual element flux may vary ± 15%

### RO membrane description and specifications

Figure 1 illustrates a schematic diagram, anatomy, and dimensions of RO membrane utilized in the removal experiment. Additionally, Table 1 contains a list of the specifications taken from its manual.

### Safety measures to handle the radioactive wastewater

To avoid contamination, the utilized radioactive wastewater was stored in a fume hood covered in two layers of plastic sheet. The stock solution was stored behind a 10-cm-thick lead break in accordance with the basic radiation protection standards of “as low as reasonably achievable” (ALARA), which aims to decrease the dose rate to the lowest possible level. Personnel protective equipments (PPE) were used, such as Latex gloves, disposable suits, overhead, overshoes, and passive (TLD) and active (EPD) dosimetry systems.

### Membrane filtration experiments

**Fig. 2:**
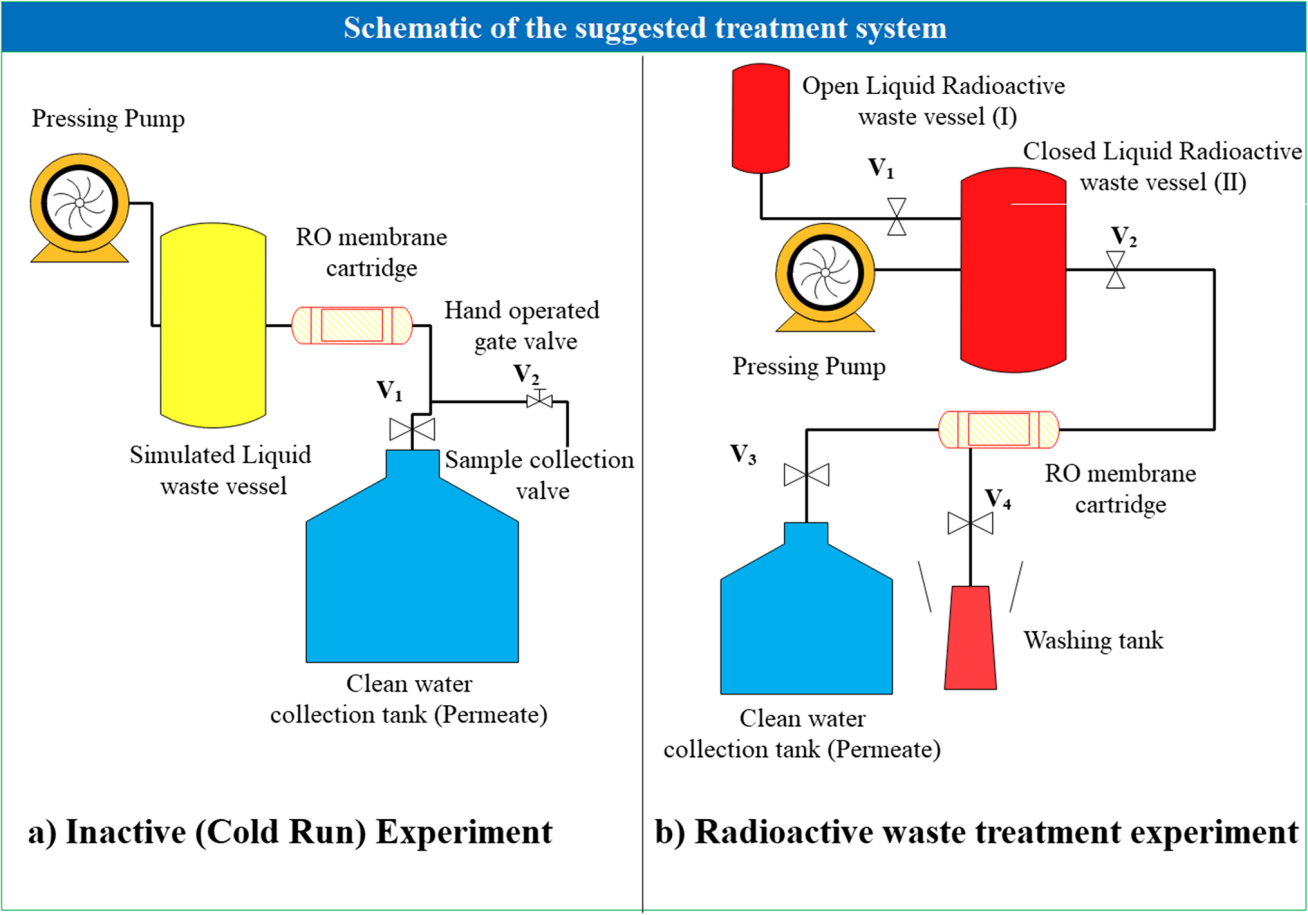
**a** Schematic diagram of the primary design of an RO-TLC membrane filtration system for inactive experiments. **b** Second design for a radioactive wastewater filtration system

Firstly, solutions of KOH and CsCl (1 mM) were prepared for inactive experiments. The pH of the CsCl solution was adjusted by the ammonia solution. The water permeability across the membrane at a pressure of 1.5 bars was evaluated using a membrane filtering system (Fig. 2a). The membrane was modified by using a mixture of solutions of various acids prior to the addition of alkaline metals. Samples that were obtained both before and after loading were measured by ICP-OES.

### Radioactive filtration experiments

In order to handle radioactive wastewater, another system was constructed in a fume hood behind a Pb shield of 10-cm thick. The glass vessel was replaced with a redesigned one that has a robust, transparent plastic wall that is capable of withstanding pressures of up to 7 bar. Before this vessel, there was another one for liquid waste collection, as illustrated in Fig. 2b. Consequently, the liquid waste was inserted into a cartridge that housed the spiral-wound membrane under pressure after being gravity-loaded into the pressured vessel. Once radioactivity was eliminated, the permeate was collected and typically loaded for two or three times. The drain outlet is constantly closed and is used only for activity stripping. Throughout the treatment, samples of permeate and feed were periodically collected, and their streams were measured. To determine the salt retention index (*R*_salt_) and the ^137^Cs retention index (*R*_Cs_) for each sample, the concentration of metal ion concentration and the radioactivity level of the ^137^Cs isotope were measured.

1 $${R}_{salt}(\%)=\frac{{C}_{f}-{C}_{p}}{{C}_{f}}\times 100$$

where *C*_*f*_ and *C*_*p*_ are metal ion concentrations for feed and permeate, respectively.

2 $${R}_{Cs}(\%)=\frac{{A}_{f}-{A}_{p}}{{A}_{f}}\times 100$$

where *A*_*f*_ and *A*_*p*_ are the ^134,137^Cs radioactivity-level for feed and permeate, respectively. The activity of the feed and permeate solution can be calculated (El Afifi et al. 2023; Hilal et al. 2022) as follows:

3 $$A\text{(Bq/L)}=\frac{\text{Net Counts - Background}}{t\times \varepsilon \times {I}_{\gamma }\times V}$$

in which (*A*) is the radioactivity level (Bq/L), *t* is the counting time, *ε* is the detector efficiency, *I*_*γ*_ is the probability of emission of each gamma ray, and *V* is the sample volume in (L).

**Fig. 3:**
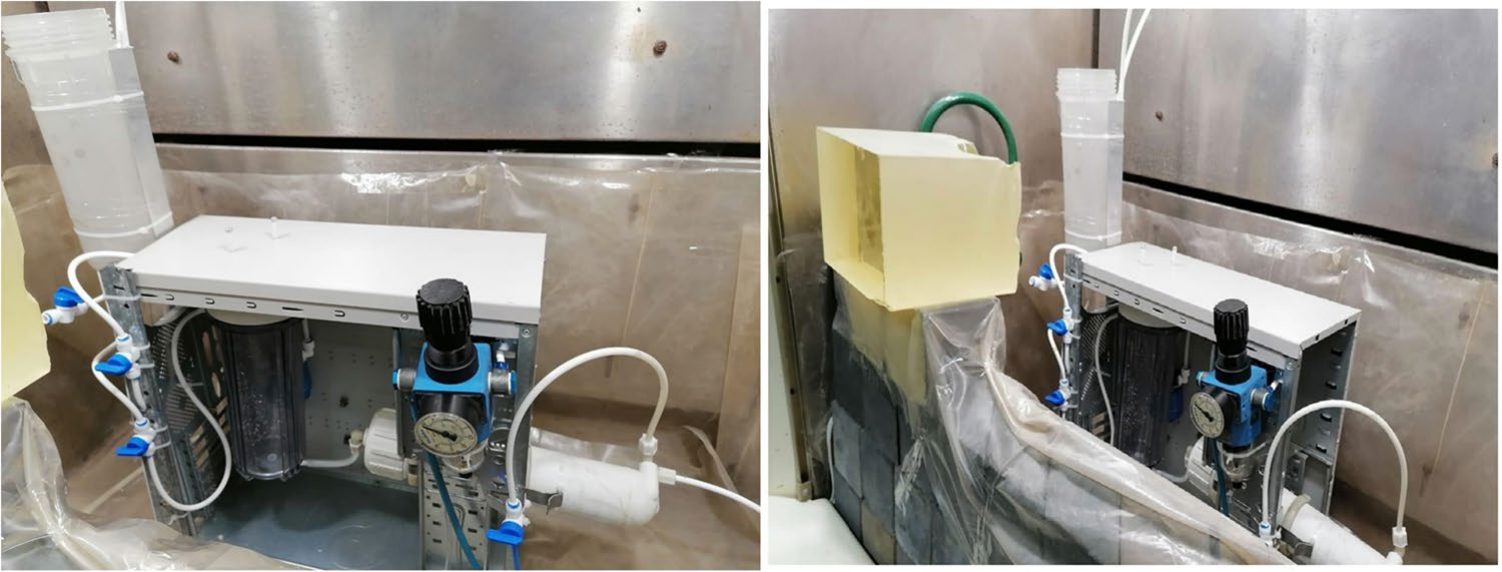
Photographs of the real radioactive filtering system that demonstrate the radiation safety measures used throughout the treatment procedure

Figure 3 displays some images from the real lab experiment using a radioactive wastewater treatment system.

### Isotherm models

The Langmuir isotherm model can be represented by the following equation:

4 $$\frac{{C}_{e}}{{q}_{e}}=\frac{{C}_{e}}{{Q}_{max}}\text{}+\text{}\frac{1}{b{Q}_{max}}$$

*Q*_max_ and *b* are the theoretical monolayer capacity (mg g^−1^) and the sorption equilibrium constant is related to the energy of sorption, respectively, and *C*_e_ is the equilibrium concentration.

A linear form of Freundlich expression can be represented as follows:

5 $$log{q}_{e}=log{K}_{F}\text{}+\text{}\frac{1}{n}log{C}_{e}$$

in which *K*_F_ and 1/n are the Freundlich constant and heterogeneity factors. It represents the amount of ^137^Cs, on the RO membrane for unit equilibrium concentration. The values of 1/n signify the deviation from the linearity of sorption as follows: (i) 1/n = 1 reflects that the sorption is linear, (ii) 1/n < 1 implies heterogeneous surface structure with minimum interaction between the adsorbed atoms, and (iii) 1/n > 1 implies homogeneous surface structure and an unfavorable Freundlich adsorption process.

## Results and discussion

### Characterization

#### Fourier transform Inferred spectroscopy (FTIR)

**Fig. 4:**
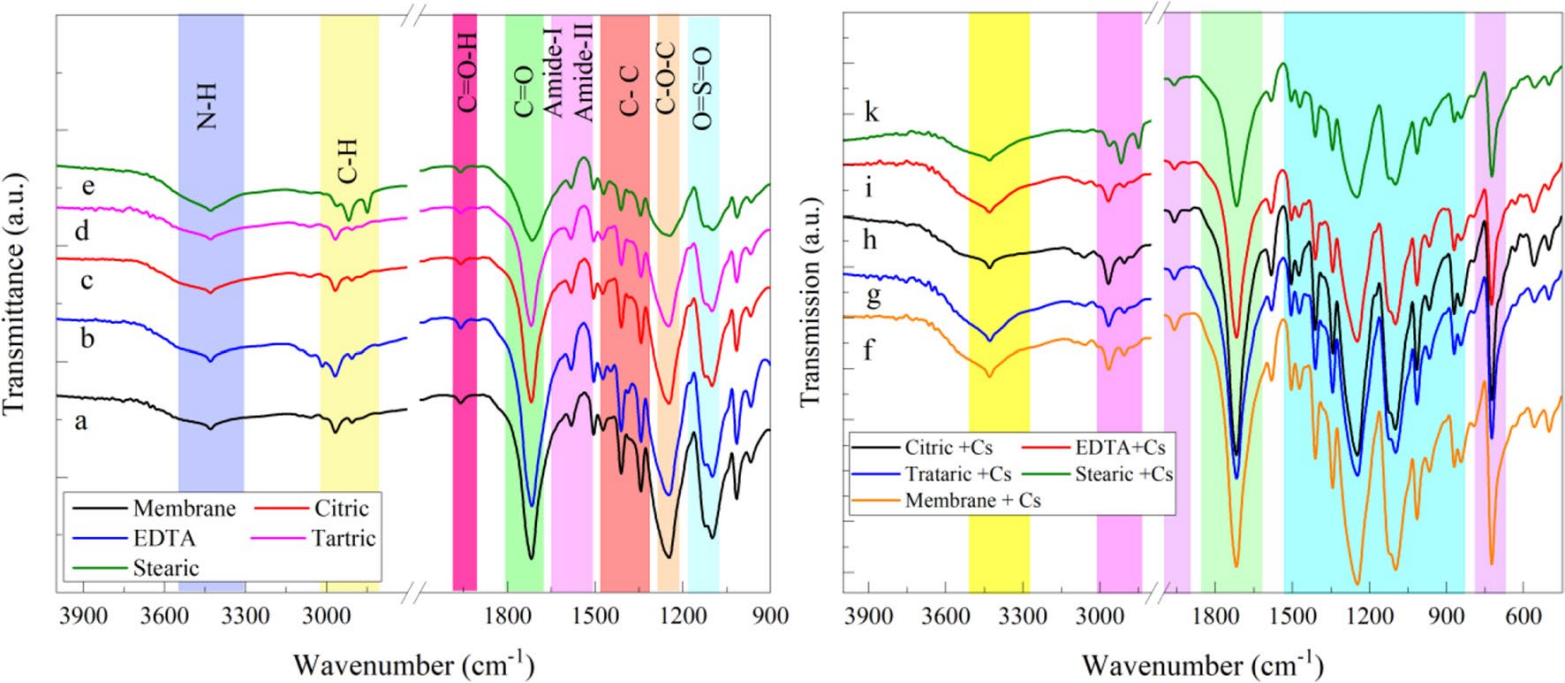
FTIR spectra for a) RO membrane, b) RO membrane modified with EDTA, c) RO membrane modified with citric acid, d) RO membrane modified with tartaric acid, e) RO membrane modified with stearic acid, f) RO membrane loaded with Cs^+^ g) RO membrane modified with tartaric acid and loaded with Cs^+^, h) RO membrane modified with citric acid and loaded with Cs^+^, i) RO membrane modified with EDTA and loaded with Cs^+^, k) RO membrane modified with stearic acid and loaded with Cs^+^

The RO membrane’s FTIR spectra before and after modification are illustrated in Fig. 4. The primary and secondary amines associated with the terminal amine groups are responsible for the -CH stretching vibration of the peak, which is observable at 2876 cm^−1^, and the equivalent -NH stretching vibration, which occurs at 3446.91 cm^−1^ (Govardhan et al. 2020). The -C=O group, amide I and II, and the bending and stretching vibrations of the carbonyl amide groups are represented by the peaks detected at 1745.64, 1672.32, and 1535.39 cm^−1^, respectively (Mostafa and El-Aassar 2012; Porubská et al. 2012). Aromatic C-C, symmetric O=S=O, ether C-O-C, and asymmetric O=S=O group stretching vibrations of the support layer of the composite membrane, respectively, are ascribed to the distinctive peaks of 1481, 1398, 1288, 1244, and 1172–1016 cm^−1^ (Tin et al. 2003; Venkata Swamy et al. 2016).

The intensity of the peaks of amides I and II was found to decrease from the original RO (Fig. 4a) to the chemically modified RO membrane (Fig. 4e), indicating the degradation of the amide group after soaking in stearic acid. The PA skin layer’s chain structure is altered and deformed as a result of this reaction, which leads to flaws in the membrane’s structure and higher permeability but lower selectivity or rejection (Govardhan et al. 2020). Finally, the use of many acids may have a combined effect that improves the sorption of ^137^Cs on the membrane, which cannot be achieved with the use of a single acid.

#### SEM and EDX

**Fig. 5:**
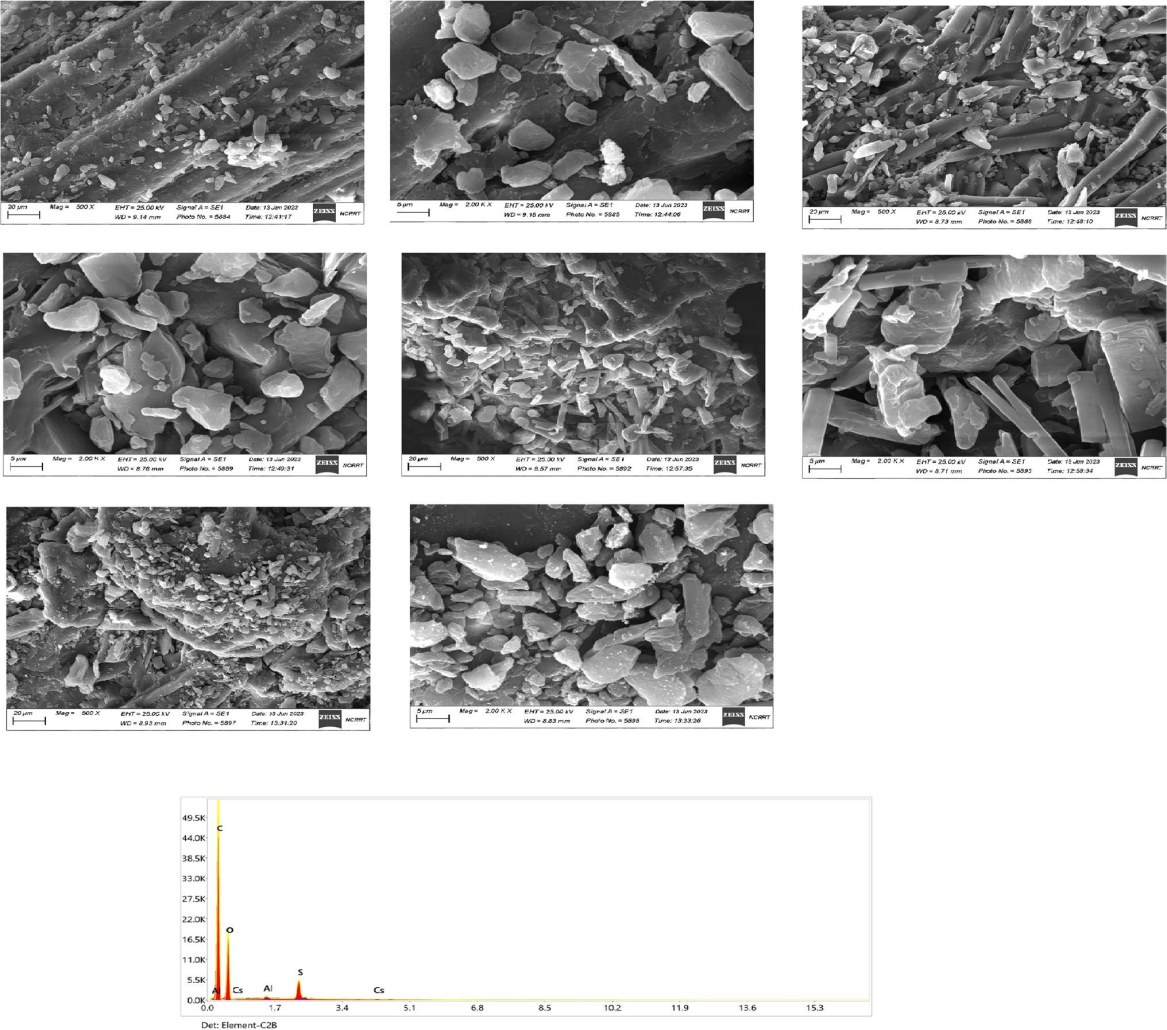
SEM and EDX results of the present work membrane samples at different magnification powers for different modifications of the RO membrane using different acids

SEM images in Fig. 5 Show a noticeable increase in the porous number on the membrane surface due to the soaking of the membrane into different acids, which may support the physical sorption of Cs^+^ metal ions on the membrane. Also, from Table 2, it is obvious that adding EDTA increased the Al and S content rather than the other acids; moreover, stearic acid increased the C and O content compared to other acids. After loading Cs^+^ metal ions, it is shown that a major increase in Cs^+^ concentration is obtained after tartaric is added, with a minor effect of EDTA and stearic acid on the Cs^+^ concentration. This may give the contribution of the added acids as a combined effect to enhance the sorption effect of the Cs^+^ metal ions on the membrane.

**Table 2:** The Results of EDX analysis of different membranes modified with different acids before and after Cs^+^ loading

Element	Percent (%)	Before Cs^+^ loading					After Cs^+^ loading				
		RO	Tartaric	Citric	EDTA	Stearic	RO	Tartaric	Citric	EDTA	Stearic
C K	Weight (%)	64.47	63.97	64.47	65.26	66.66	63.2	62.14	62.82	60.87	66.6
O K		34.08	34.83	34.08	32.45	32.59	35.06	36.28	35.93	38.46	32.09
Al K		0.16	0.15	0.16	0.18	0.14	0.22	0.11	0.13	0.15	0.14
S K		1.28	1.05	1.28	2.11	0.61	1.36	0.94	1.03	0.42	1.09
Cs L		––	––	––	––	––	0.15	0.54	0.08	0.09	0.07
C K	Atomic (%)	71.16	70.63	71.16	72.12	72.92	70.11	69.18	69.61	67.65	73.05
O K		28.23	28.86	28.23	26.92	26.76	29.19	30.32	29.89	32.09	26.42
Al K		0.08	0.07	0.08	0.09	0.07	0.11	0.05	0.07	0.08	0.07
S K		0.53	0.44	0.53	0.88	0.25	0.57	0.39	0.43	0.17	0.45
Cs L		––	––	––	––	––	0.02	0.05	0.01	0.01	0.01

### System optimization and procedures

The RO membrane of interest was tested at a pressure of 1.5 bars three times with water devoid of metal ions to estimate its permeability, resulting in equal quantities at the entrance and outflow prior to being exposed to an ion-bearing solution.

**Fig. 6:**
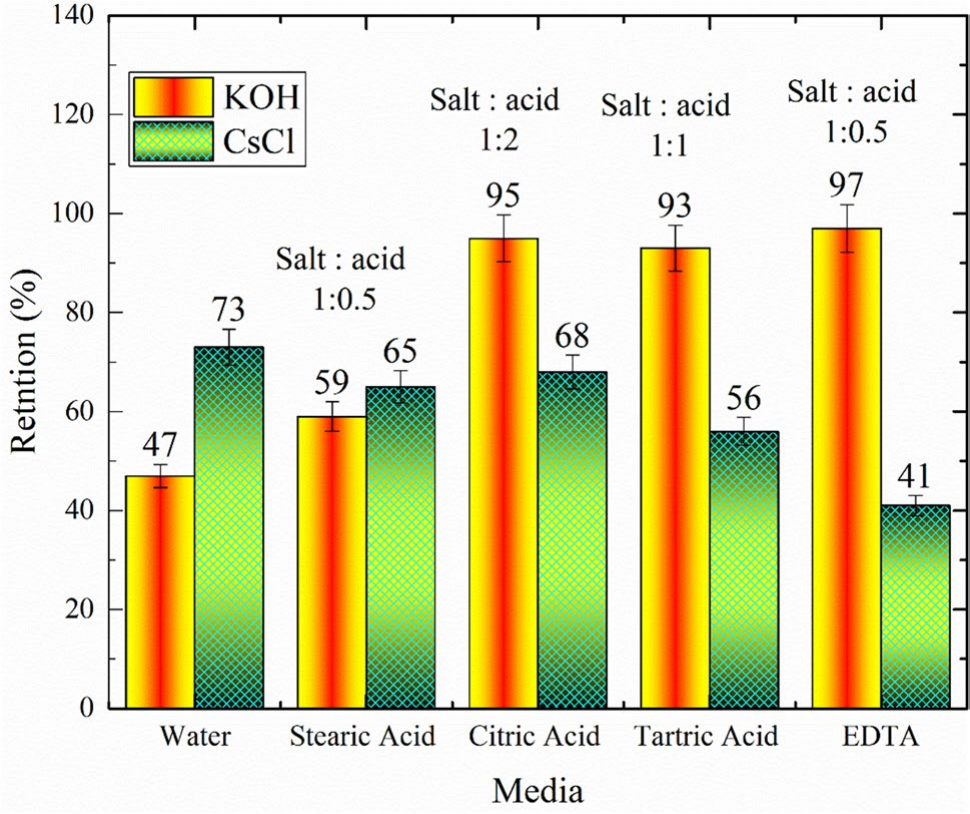
A Preliminary study to test the performance of the RO membrane in the retention of K^+^ (as KOH) and Cs^+^ (as CsCl) metal ions in water and with different ratios of acids

Depending on the specification of the used RO membrane, its rejection ability to K^+^ was almost equal to 98% at 500 ppm softened tap water at 4.5 bar, and 25 °C; with the similarity between K^+^ and Cs^+^ alkaline metal ions, a preliminary experiment was conducted using a separately 1 mM of CsCl and KOH in water and with different ratios from citric acid, stearic acid, tartaric acid, and EDTA media. The obtained results are shown in Fig. 6. It is obvious that the retention of K^+^ is higher than Cs^+^ metal ion at different media and salt-to-acid ratios. The maximum retention was obtained at (1:0.5) KOH: EDTA. This can indicate that the retention is depending on the salt media (chloride or hydroxide) forms.

The majority of radioisotopes discovered in the liquid waste in the aqueous phase are ^134+137^Cs, and the pH estimate is over 12, indicating that radioactive cesium species are most likely to exist in the hydroxide form. As a result, by converting CsCl into CsOH, it behaves similarly to KOH and the retention will be increased. The following procedures were examined to convert CsCl into CsOH:

- Electro-hydrolysis
- Reaction with citric acid
- Reaction with ammonia

**Fig. 7:**
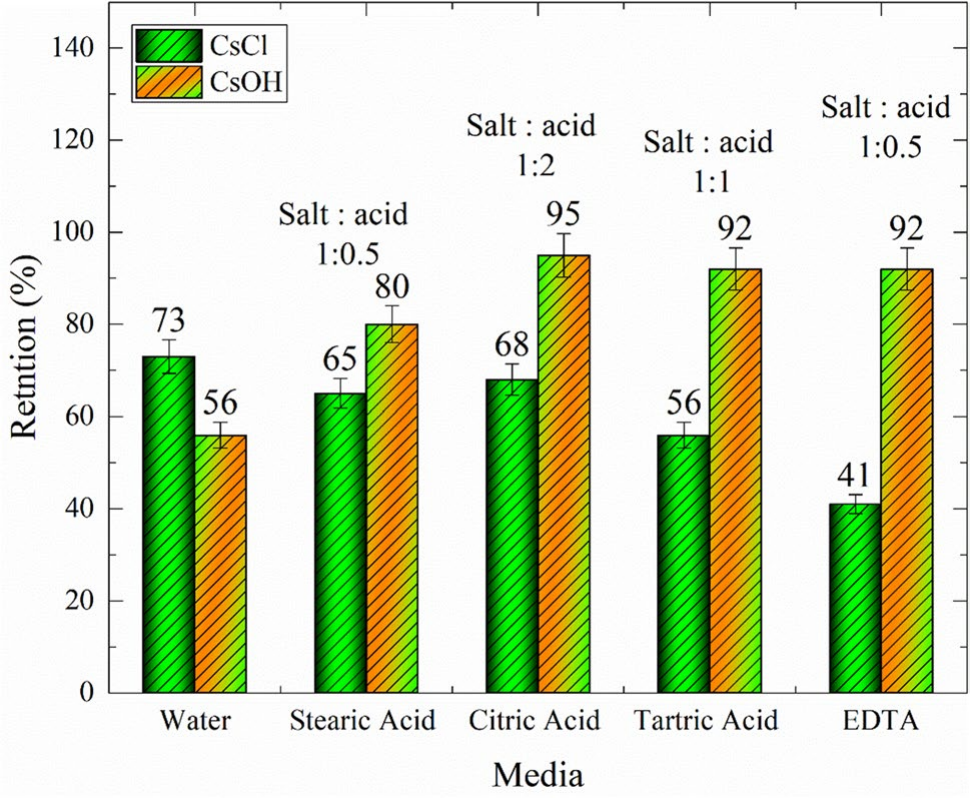
A Preliminary study to test the performance of the RO membrane in the retention of Cs^+^ in (CsOH) and (CsCl) metal ions at different ratios of acids to salt contents

The percentages of CsOH produced by electro-hydrolysis, citric acid, and ammonia were 70, 68, and 95%, respectively. To compare the performance of the RO membrane in the retention of CsCl and CsOH, the procedures of the preliminary study were carried out again. The results are presented in Fig. 7, which reveals that the conversion of CsCl into CsOH has a significant impact on the retention process. The maximum obtained retention ratio was at the (1:2) salt-to-acid ratio of citric acid with a relatively small difference when using tartaric acid and EDTA with (1:1) and (1:0.5) ratios, respectively.

Table 3 involves the data of salt retention index for both potassium and cesium from (0.001 M) concentration in the absence (pristine) and in the presence of different acids with a ratio of 1:1 (metal ions to acid) except for stearic acid (1:0.5) because its concentration is higher than (0.5 M); it became more viscous and hinders the flow of feed solution.

**Table 3:** The rejection percentage of either K^**+**^ ions or Cs^**+**^ ions from solutions containing K^**+**^ or Cs^**+**^ ions plus each acid separately

Concentration (1 mM)	Metal ions/acid ratio	Rejection percentage (%)		
		KOH	CsCl	CsCl + Amm*^#^
Pristine**	–	47	73	56
Stearic acid	1:0.5	59	65	80
Tartaric acid	1:1	82	56	92
Citric acid	1:2	91	68	95
EDTA	1:0.5	97	41	92

*Amm ammonia solution (few drops), **Only KOH or CsCl solution, and #pH higher than 12

Moreover, Cs^**+**^ ions in a solution at a pH higher than 12 have the same trend as those of K^**+**^ ions in a KOH solution. In the pure CsCl solution at pH ≤ 7, the *R*_*salt*_ decreased with the increasing number of carboxyl groups in the acid, consequently resulting in the EDTA showing a rather low retention index for cesium.

### Radioactive effluent treatment

**Fig. 8:**
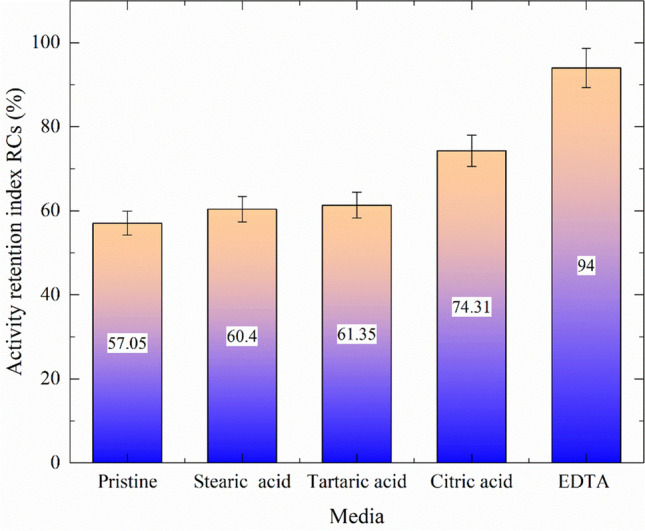
The retention index of ^137^Cs-radioactivity *R*_*Cs*_ in the absence (pristine) and in the presence of the investigated acids

Using radioactive waste that predominantly contained ^137^Cs, four acids of interest were studied. In the absence (pristine) and with stearic acid, tartaric acid, citric acid, and EDTA, Fig. 8 illustrates the retention index of Cs-activity *R*_Cs_.

All these acids were tested at the same concentration, with approximately equal amount of radioactivity for each acid. It is observed that EDTA has the highest retention factor for ^137^Cs removal from feed solution, followed by citric, tartaric, and stearic acids, which have a lower retention index.

**Fig. 9:**
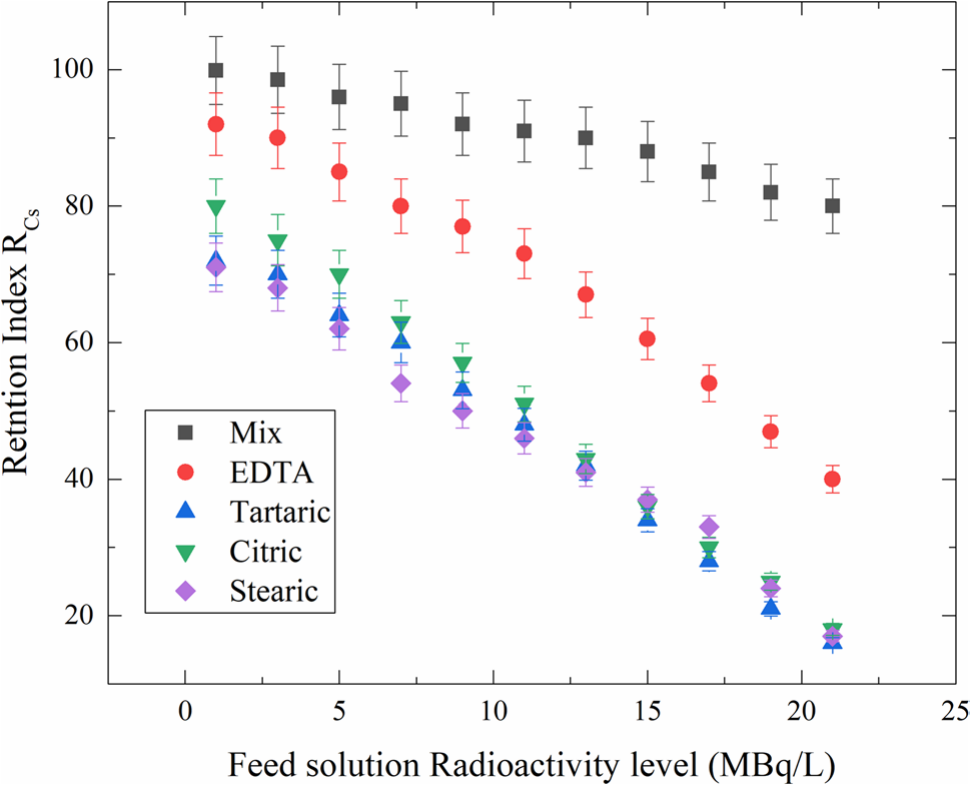
The retention index of ^137^Cs as a function of feed radioactivity level in (MBq/L)

Based on the characterization results and the preliminary study, it can be suggested that adding the selected acids can increase the retention index of ^137^Cs on the RO membrane. Therefore, it is recommended to use the present work acids mixture, 0.5 mM stearic acid, 1 mM tartaric acid, 2 mM citric acid, and 0.5 mM EDTA as a modification for the RO membrane before using in the ^137^Cs removal from wastewater. To test this assumption, the retention index for each acid and the mixture of them were calculated as a function of the feed solution activity (Fig. 9).

As cleared from the figure, the maximum retention index was achieved by modifying the RO membrane using the optimal concentrations of the chosen acids. In addition, while using high feed concentrations, the retention index value does not decrease to lower values as it does with other modification media. The result confirms the positive result of the suggested modification for the RO membrane in the removal of ^137^Cs form radioactive wastewater streams.

**Fig. 10:**
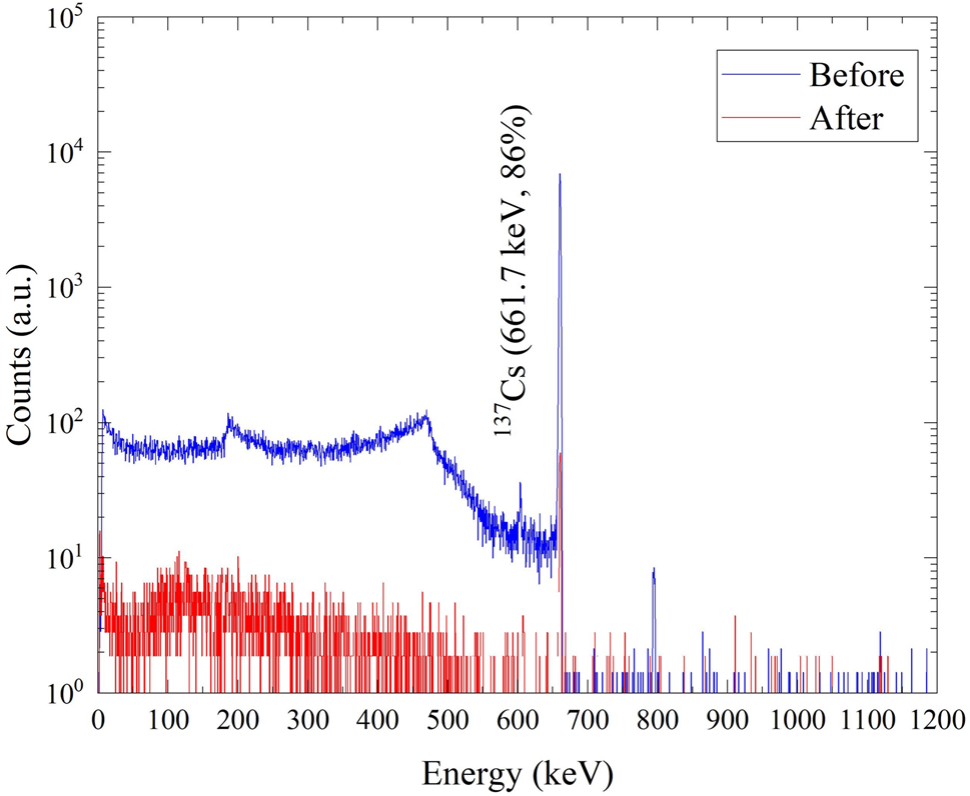
Gamma spectrum of the feed and permeate solution after ^137^Cs removal

The measured gamma spectrum of the wastewater before and after ^137^Cs removal is plotted in Fig. 10.

### Cs-activity elution

**Fig. 11:**
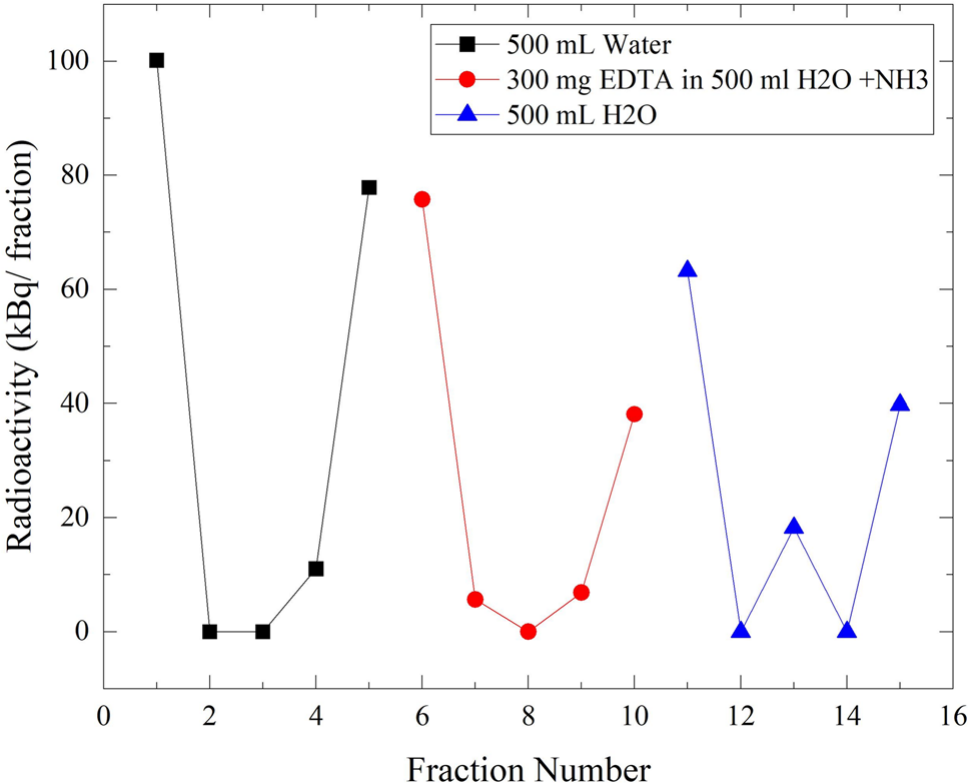
The Elution curve of ^137^Cs-radioactivity piling on the membrane from permeate let

The elution of the radioactivity that is loaded on the membrane is an important issue for the membrane regeneration process. Several trials have been performed to elute Cs-activity already there on the membrane using different eluents, as illustrated in Fig. 11. The first trial was with 500 mL of water, the second was with 500 mL of EDTA solution and ammonia, and the third was with another 500 mL of water. All fractions collected from the permeate outlet were free of activity, with the exception of those obtained at the beginning of pressure load and pressure loss. This could be mechanically explained in such a way that the permeate outlet in the membrane inside its housing is loose, which allowed some activity to pass illegally through the permeate outlet. This feature demonstrated that there was some activity at the beginning of each test.

**Fig. 12:**
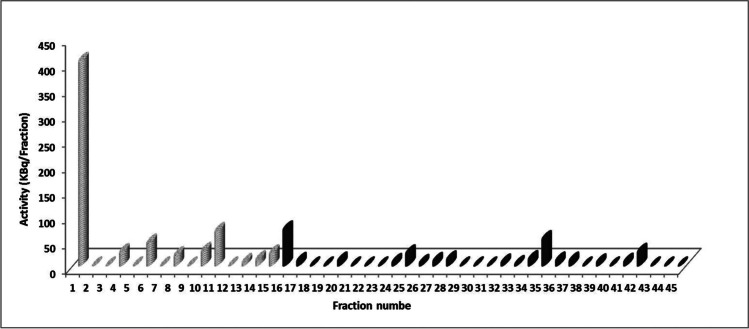
The elution curve of Cs-activity piling on the membrane from overflow let using 1.5 L of ammonia solution followed by 3 L of water

According to the feature shown in Fig. 7, the Cs-activity in the pristine solution has a low retention index (*R*_Cs_ = 56%). This led to additional attempts to elute the activity as given in Fig. 12, where 1.5 L of diluted ammonia was applied in a way of 1 L followed by 0.5 L. Only the first fraction contains some activity but is rather low (450 kBq/fraction). In addition, 3 L of water was applied; however, eluting the retained activity on the membrane is not feasible. It is important to mention that in these experiments, the overflow outlet was always closed.

**Fig. 13:**
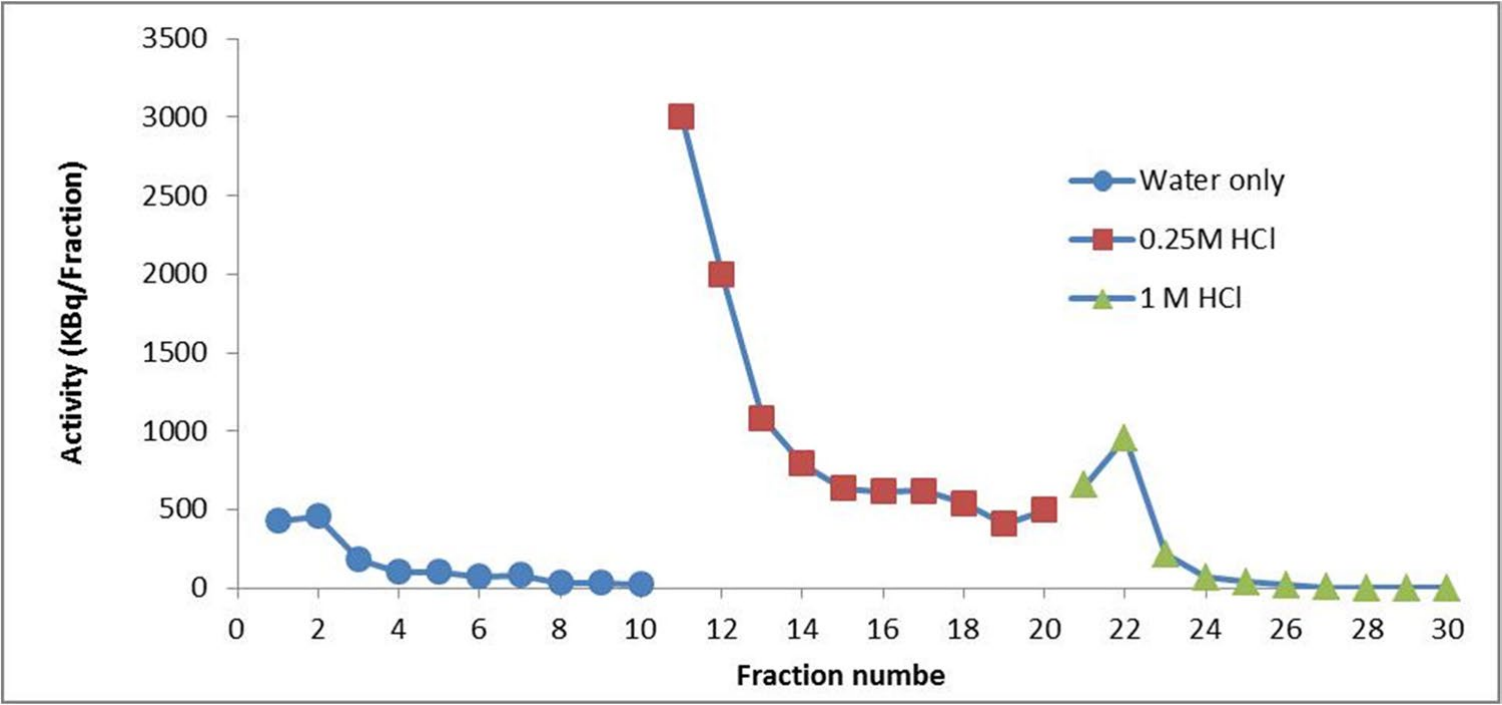
The elution curve of Cs-activity piling on the membrane from overflow let using water, 0.25 M HCl, and 1 M HCl in sequence

For the activity Elution, it is required to close the permeate outlet and allow the overflow outlet to be opened. Despite it being likely easy to displace the activity piling on a surface of the TLC membrane of interest to pass through the overflow outlet, the water volume of 1 L was not sufficient to displace the Cs-activity. During the elution, the 1st and 2nd fractions contain a low level of activity, as shown in Fig. 13, as well as the other fractions have no activity, which means the CS-activity is not as a pile, contrary to the existence as a homogenously distributed and a specifically adsorbed on a surface of the membrane. Thus, the non-specific adsorbed activity goes out when water flows. Therefore, the plain water was replaced by diluted mineral acid of 0.25 M HCl to overcome the specific adsorption feature of the Cs-activity. The volume of 1 L of this concentration passed through the membrane, which strongly displaced the most activity in the first 3 fractions. On the other hand, although the advantage of displacement of the activity in 300 mL the other fractions of the same eluent still have the appreciable Cs-activity of about the same activity in each fraction, this means this concentration is not sufficient to elute the Cs-activity in a small volume not more than 300 mL. Therefore, a status that considers the HCl as an eluent is consistent with the purpose of eluting the Cs-activity in a small volume.

As shown in Fig. 13, 1 M HCl was also investigated as an eluent to displace the activity; 200 mL of this concentration is sufficient to eliminate all residual activity from the membrane surface. It is concluded that the 300 mL of 1 M HCl could be sufficient for Cs-activity elution from the TLC membrane surface, making it applicable for further treatment of liquid waste as well as the activity in a small volume to be useful in industrial purposes.

### Isotherm models

**Fig. 14:**
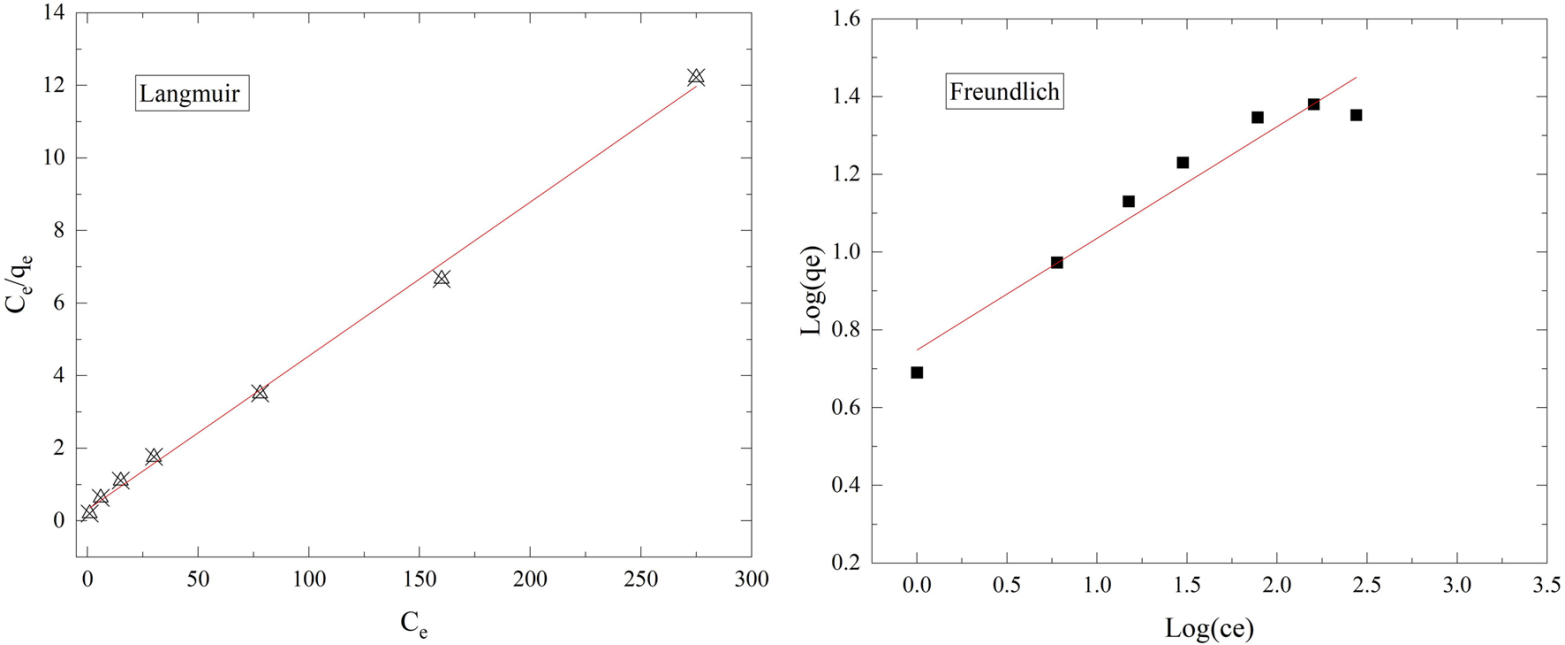
Langmuir and Freundlich isotherm models

Plotting *C*_e_/*q*_e_ and *C*_e_ gives straight lines for ^137^Cs onto RO membrane sorbent at temperatures of 298 K, as shown in Fig. 14, confirming that the Langmuir isotherm is applicable and a reasonable representation of the chemisorption isotherm. Table 4 displays the data calculated from the slopes and intercepts of the linear form of the Langmuir model for ^137^Cs sorption onto the RO membrane at 298 K. The data presented in Table 4 are *R*^2^ = 0.996 and the *Q*_max_ was 23.56 mg g^−1^.

**Table 4:** Isotherm model for Cs + adsorption on RO membrane

Isotherm model	Parameter	Cs^+^ cation	Temperature (K)
Langmuir	*Q*_max_ (mg g^−1^)	23.568	298
	*b* (L mg^−1^)	0.138	
	*R*^2^	0.9964	
Freundlich	1/n	0.2873	
	*k*_f_	2.113	
	*R*^2^	0.9338	

## Conclusion

RO-TLC membrane, after being conditioned by various acids, is applied for the removal of radio cesium. Different acids were essentially used in the feed solution, namely, monobasic acid and multi-basic acid; stearic, tartaric, citric acids; and EDTA. The obtained results reveal that EDTA is the most suitable acid for Cs removal from feed solution because of its highest *R*_*Cs*_; besides, it is easily soluble at a high pH of 12. Using EDTA as a modifier, about 94.5% of radio cesium from radio waste was rejected on the membrane, which is close to that of the cold experiment (97%), as well as the ^134^Cs-activity, which was rejected completely on the membrane at the first run. It is clarified that four runs were sufficient to reject the most of Cs-activity on the membrane, about 97.5% of the initial activity; however, further treatment is needed to overcome the residual activity of about 2.5%. In general, not only one technique is not sufficient to achieve the complete removal of Cs-activity, but a combination of more than one technique is also needed to achieve the complete removal of Cs-activity from liquid waste.
